# The Value of Native Plants and Local Production in an Era of Global Agriculture

**DOI:** 10.3389/fpls.2017.02069

**Published:** 2017-12-05

**Authors:** Oren Shelef, Peter J. Weisberg, Frederick D. Provenza

**Affiliations:** ^1^Biology Department, University of Nevada, Reno, Reno, NV, United States; ^2^Department of Natural Resources and Environmental Science, University of Nevada, Reno, Reno, NV, United States; ^3^Department of Wildland Resources, Utah State University, Logan, UT, United States

**Keywords:** regenerative agriculture, local food, domestication, plant utilization, *Pinus monophylla*, *Pinus edulis*

## Abstract

For addressing potential food shortages, a fundamental tradeoff exists between investing more resources to increasing productivity of existing crops, as opposed to increasing crop diversity by incorporating more species. We explore ways to use local plants as food resources and the potential to promote food diversity and agricultural resilience. We discuss how use of local plants and the practice of local agriculture can contribute to ongoing adaptability in times of global change. Most food crops are now produced, transported, and consumed long distances from their homelands of origin. At the same time, research and practices are directed primarily at improving the productivity of a small number of existing crops that form the cornerstone of a global food economy, rather than to increasing crop diversity. The result is a loss of agro-biodiversity, leading to a food industry that is more susceptible to abiotic and biotic stressors, and more at risk of catastrophic losses. Humans cultivate only about 150 of an estimated 30,000 edible plant species worldwide, with only 30 plant species comprising the vast majority of our diets. To some extent, these practices explain the food disparity among human populations, where nearly 1 billion people suffer insufficient nutrition and 2 billion people are obese or overweight. Commercial uses of new crops and wild plants of local origin have the potential to diversify global food production and better enable local adaptation to the diverse environments humans inhabit. We discuss the advantages, obstacles, and risks of using local plants. We also describe a case study—the missed opportunity to produce pine nuts commercially in the Western United States. We discuss the potential consequences of using local pine nuts rather than importing them overseas. Finally, we provide a list of edible native plants, and synthesize the state of research concerning the potential and challenges in using them for food production. The goal of our synthesis is to support more local food production using native plants in an ecologically sustainable manner.

## Regenerative agriculture in a global economy

Feeding growing populations with increasing demands for quality, healthy, savory, and attractive food is a vital challenge for humanity. Contemporary agricultural practices have endeavored to do so by improving productivity of a small number of existing crops, rather than by increasing crop diversity. Developing new crops and learning to use wild plants creates the potential to diversify global food production and better enable local adaptation to the diverse and changing environments humans inhabit (Provenza, 2008). Manifestations of global changes—climatic, ecological, behavioral, and technological—emphasize the need to improve food production in ways that reduce negative impacts on the carrying capacities of the ecosystems we rely upon to sustain us. Regenerative-ecological agriculture can restore earth and human health through the five processes that enable and link all life: flow of energy, captured by plants through photosynthesis; soil-mineral cycles that provides nutrients for life; the water cycle essential for life; ecological relationships that create soil-plant-animal communities; and human-land linkages including landscape-genomics and our dialogue with nature (Massy, 2017). As part of those essential linkages, we could also benefit from re-learning to use local plants as sources of healthy food and other products, with attention and concern for environmental issues. Humans have used plants in many ways that include various forms of domestication, gathering, horticulture (Harris and Fuller, 2014), aquaculture and production of secondary products like grazing (livestock, bees) and forestry. While the use of animals for food and other products also has a fundamental role in agriculture, in this review we focus on plant-based agriculture.

Shelef et al. (2018) describe four aspects of sustainable agriculture: land management, resource management, the human interface, and the ecosystem interface. They argue that using native plants as part of local food production can help create more sustainable agriculture. While local food production has attracted much attention recently, use of native plants in local food production has received little attention. Most food crops are produced, transported, and consumed long distances from their location of origin. Moreover, according to the Food and Agriculture Organization of the United Nations (FAO), more than 90% of the calories humans consume come from just 30 plant species (Hammer et al., 2003). We cultivate only about 150 out of an estimated 30,000 edible plant species (Sethi, 2015 and references within). Within these few species, genetic diversity has decreased as the number of marketed varieties has shrunk. For example, out of more than 7000 varieties of apples grown in the United States in the last century, over 6000 varieties have become extinct (Shand, 2000). At the same time, research efforts focus primarily on improving productivity of a few existing crop species, rather than increasing crop diversity. This represents a serious loss of agro-biodiversity and erosion of genetic diversity, leading to a food industry and human populations more susceptible to stressors associated with global environmental change. Sethi (2015) described the potential loss of food diversity in detail and the FAO estimates there has been a 75% reduction in crop diversity globally.

In this review, we discuss the tradeoffs between efforts to improve the productivity of a limited number of crops and efforts to increase crop diversity by recruiting new species and using local species. We describe the concepts of local agriculture and use of native species, elaborating on the ways these concepts are perceived today. Commercial uses of new crops and wild plants have potential, through diversification, to make global food production more sustainable and resilient. We discuss the advantages, obstacles, and risks associated with using local plants. We also provide a case study—the missed opportunity to utilize locally produced pine nuts at large scale in the Western United States. Finally, we provide a list of consumable native plants, and analyze research endeavors to study them.

In the process of using plants over thousands of years humans have influenced plant evolution (Harris and Hillman, 1989). The early days of agriculture began about 10,000 years ago (Zohary et al., 2012), when people used local species and selected for desirable traits for human consumption (Diamond, 2002). Domestication began with the cultivation of wheat in the Fertile Crescent and rapidly spread throughout Europe (Zohary et al., 2012). Once domesticated, many crops expanded rapidly and are now used in areas where they did not originate (Drewnowski and Popkin, 1997). To a large extent, this is the case with the seven most globally used food crops: rice (*Oryza sativa*), wheat (*Triticum aestivum*), soybeans (*Glycine max*), sugarcane (*Saccharum* spp.), tomato (*Solanum lycopersicum)*, maize (*Zea mays*), and potato (*Solanum tuberosum*) (FAO, 2016). In the United States, nearly all of the plants people consume are exotic species, such as corn, rice, wheat, and soybeans (Pimentel et al., 2005). Most research is now devoted to improving existing crops through artificial selection and breeding, agro-technical approaches and genetic modifications (Lemaux, 2009). New crops developed from local species are the exception (Shelef et al., 2016). Intensive agricultural practices developed to increase yield are associated with ecological and environmental costs that include reducing biodiversity, accelerating land degradation, applying fertilizers, contaminating water and spreading pesticides hazardous to human health (Horrigan et al., 2002; Massy, 2017). Future agriculture will have to cope with increasing food demands for greater populations in the face of changing climates, including changes in the frequency and intensity of precipitation, increasing occurrence of droughts (Howden et al., 2007), and increasing use of chemicals (Boxall et al., 2009). Developing new plant varieties for crop production can help mitigate these challenges by increasing the opportunity to match local crop species with changing environmental conditions.

## What we talk about when we talk about local agriculture

Local agriculture has two facets. One is use of native plant species that often have not been studied or commercialized. The other is food production, which involves a short distance life cycle from field to plate. Shorter cycles between production and consumption reduce carbon footprints, defined as the equivalent tons of CO_2_ emissions produced by a particular set of activities. Food miles (Smith et al., 2005), the distance of food transport, is a critical factor determining the carbon footprint of food production. Edwards-Jones et al. (2008) criticized the popular assumption that “local is better,” arguing that most analyses lack the empirical evidence needed for explicit life-cycle assessment. For example, they contend the distance considered within the range of “locality” is ambiguously interpreted, and criticize the widespread reliance on supply-chain-distance as the sole metric for evaluating food quality. They also question other ways we attempt to assess the nutritional quality and value of food. Their arguments highlight some weaknesses of the “local is better” assumption that we consider later. We stress that the important conceptual part of local plant consumption is the one that is usually least discussed—the use of native plants for novel agriculture.

**Figure 1:**
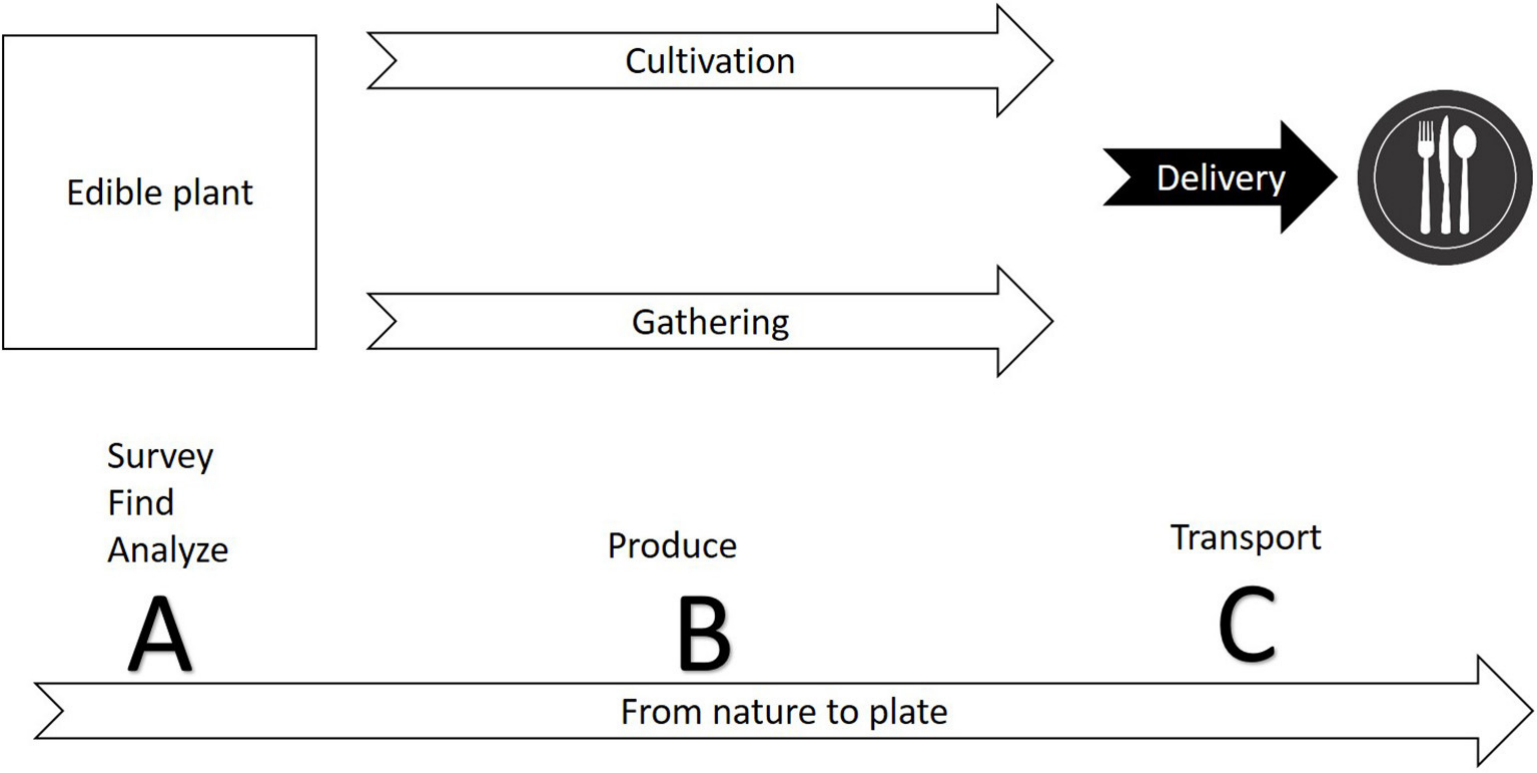
A conceptual diagram illustrating the production cycle of edible plants from nature to plate. Commercialization of a new plant involves three phases: **(A)** finding a species with potential for safe use commercially—the species may be used already by indigenous people or may be totally novel; **(B)** establishing the technique for production through cultivation or gathering; **(C)** developing ways to harvest, store, and deliver the crop.

The first step in commercializing any plant species is the search for relevant plants (Figure 1). The FAO estimates a mere 1% of available tree species have been studied for agricultural potential. As a matter of practical consideration, it is easier to search for agricultural potential under the bright light of traditional cultures. Ethnobotany, the study of native plant uses through the traditional knowledge of a local culture (Balick and Cox, 1996), had a significant contribution to the use of plants in the modern society, mainly for the pharmaceutical industry (Snader and McCloud, 1994). Ethnobotany uses socio-botanical surveys and questionnaires as a first step prior to phytochemical inspection. This practice is sometimes criticized for relying more on “primitive conception” through qualitative sociology inquiry than on “hard sciences” such as phytochemistry, pharmacology and agronomy. The search for new drugs is the main economic driver behind ethnobotanical studies, but increasing agrodiversity is as important as developing new drugs. Nevertheless, ethnobotanical studies have revealed important knowledge about native plants as food resource. Worth mentioning is a book by Daniel Moerman (1998) who listed the ten plants most commonly used for food by Native Americans: Common chokecherry (*Prunus virginiana*), Banana yucca (*Yucca baccata*), Saskatoon serviceberry (*Amelanchier alnifolia*), Honey mesquite (*Prosopis glandulosa*), Saguaro (*Carnegiea gigantea*), Broadleaf cattail (*Typha latifolia*), Corn (*Zea mays*), American red raspberry (*Rubus strigosus*), Salmonberry (*Rubus spectabilis*), and Thimbleberry (*Rubus parviflorus*). It is also worth mentioning that of all these plants, only the last four (corn and the three berries) are commercially used today in considerable scale. For additional examples of edible plants of the new world, and potential obstacles for commercialization, see Table 1.

**Table 1:** Examples of some edible plants of the new world, main consumption practice and possible reasons for commercialization hurdles.

**Common name**	**Latin name**	**Origin**	**Consumed parts**	**Consumption**	**What challenges Commercialization**	**References**
Açaí palm	*Euterpe oleracea*	Northern South America	Fruit and fruit pulp	Juice and juice as additive	Irregular supply, inconsistent quality, lack know-how in cultivation and processing	Pacheco-Palencia et al., 2007
Agave	*Agave tequilana*	Mexico, Southwestern United States	*Piña* (core)	Tequila, sweetener, potential biofuel	Traditionally grown in Mexico, local know-how and plant adaptations hinder wide distribution	Nobel, 1994; Davis et al., 2011
Banana yucca	*Yucca baccata*	South Western United States	Fruit	Fresh or heated		Moerman, 1998
Berries: Huckleberry, Raspberry, Salmonberry, Saskatoon serviceberry, Thimbleberry	*Vaccinium* spp., *Rubus strigosus, Rubus spectabilis, Amelanchier alnifolia, Rubus parviflorus*	North America	Fruits	Raw, dried, or as juice and jam	Yield fluctuations, cultivation barriers, fire management. Some cultivation developed in the last decade	Moerman, 1998; Barney, 2003
Biscuitroot	*Lomatium* Spp.	Western North America	Roots	Starchy food, cooked or grained to flour		Herzog, 2014
Bitterroot	*Lewisia rediviva*	Western North America	Roots	Traditional delicacy		Bandringa, 1999
California Black Oak (among other species)	*Quercus kelloggii*	Western United States	Acorns	Staple food for direct consumption, flour, oil	Long generation time, harvest is hard, expensive labor, process is needed (dry roasting, grinding, press)	Wolf, 1945; Ocean, 1993
Cassava	*Manihot esculenta*	West-central Brazil	USO	Starch for flour	Palate preferences limit it mainly to the southern hemisphere	Caballero-Arias, 2015
Chokecherry (bitter-berry)	*Prunus virginiana*	North America	Fruit	Cooked jelly, jam, syrup, and wine	Hard to collect or domesticate, need processing	Moerman, 1998
Common Sunflower	*Helianthus annuus*	North America	Seeds, flower bud	raw, roasted, cooked, dried, and ground, oil, coffee substitute	Commercially used	Heiser, 1976
Honey mesquite	*Prosopis glandulosa*	Southwestern United States and Northern Mexico	Pods	Starchy flour		Moerman, 1998
Indian rice grass	*Oryzopsis hymenoides*	North America	Seeds	Flour	Cultivated as Gluten-free grain	Dunmire and Tierney, 1997; Moreno et al., 2014
Joshua Tree	*Yucca brevifolia*	Arid southwestern United States	Seeds, flower buds	Oil	Product is not attractive to justify cultivation. Low indexes of cultural significance Stoffle et al., 1990	Wolf, 1945
Mushrooms	*Tricholoma magnivelare, Marasmius oreades, Lycoperdon sp., Agaricus sp., Tremellodon sp., Latarius deliciosus, Lycoperdon perlatum, Morchella sp., Pleurotus sp., Ramaria sp*.		Sporocarp (fruit body)	Food, medicine	Lack of knowledge on gathering patterns, gathering systems are laborious	Richards, 1997
Pinweed	*Erodium cicutarium*	North America	Entire plant			Lovell, 1926
Pinyon Pine nuts	*Pinus monophylla, P. edulis, P. quadrifolia, P. remota, P. culminicola, P. johannis, P. orizabensis, P. cembroides*	New world	seeds	Raw food, pesto industry, oil,	Long generation time, harvest is hard, expensive labor, cheaper substitutes, seed extraction	Sharashkin and Gold, 2004
Saguaro	*Carnegiea gigantea*	Southwestern United States and Northern Mexico	Fruits	Fermented drink		Moerman, 1998
Sego lily	*Calochortus nuttallii*	Western United States	USO (underground storage organs), seeds, and flowers	starchy grain	Cultivated as ornamental plant, slow maturation of bulbs	Herzog, 2014
Wild onions and garlic	*Allium spp*.	Northern Hemisphere	Roots, leaves	Direct or cooked	Of hundreds species only handful are cultivated	Rabinowitch and Currah, 2002

*An exhaustive list of all edible plants is out of the scope of this paper. Organized by alphabetically order of the common names*.

*We decided to focus in the New World, where agriculture history stretched back only several decades. Farmers in the Americas could chose to develop local plants, or utilize crops they imported from the Old World. This short history is emphasizing the tendency to prefer a limited number of local or imported crops rather than expanding agrodiversity by using local plants*.

**Figure 2:**
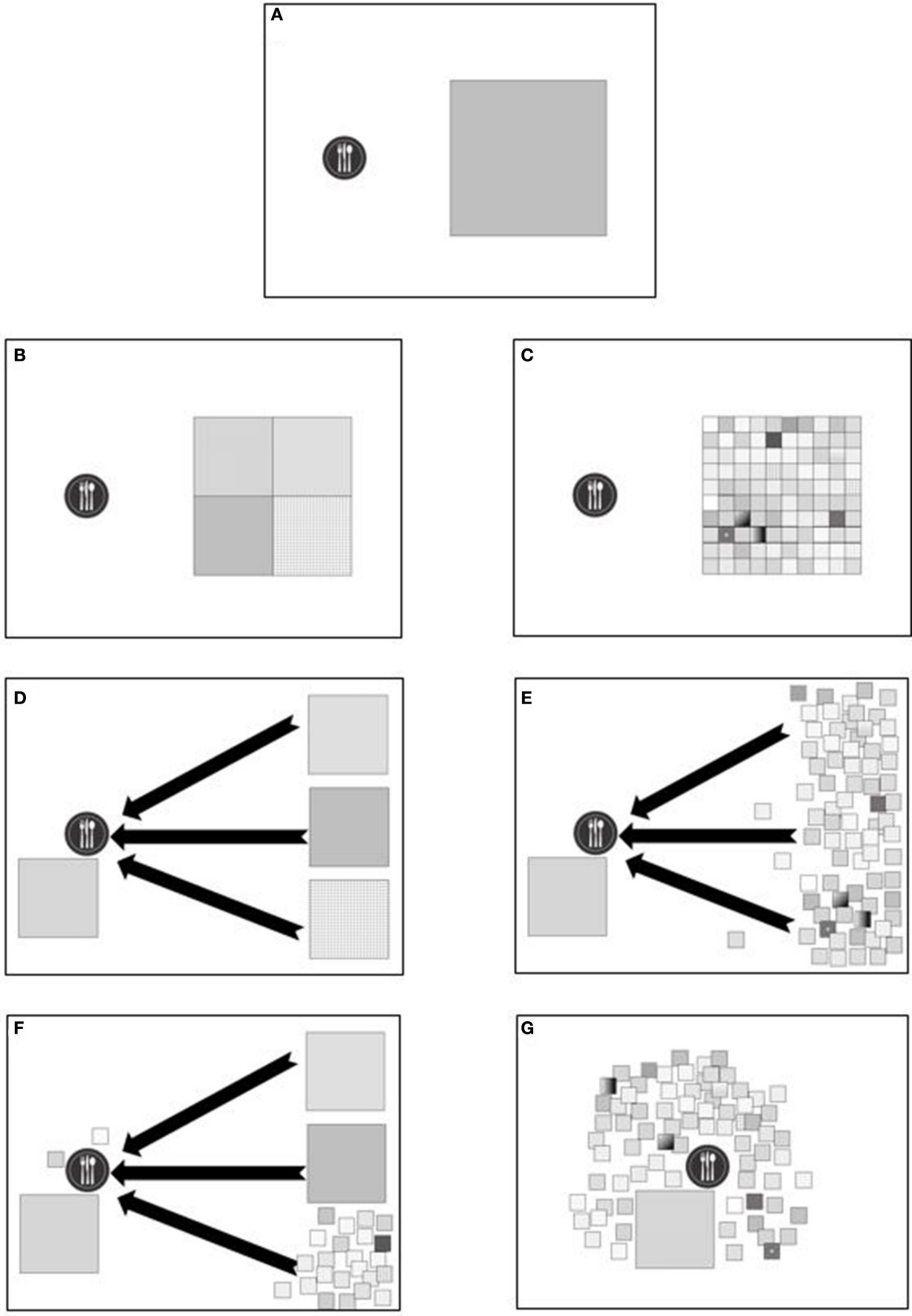
Conceptual illustration of local food production, native plants and agro-diversity. Illustrations **(A–G)** describe the differences between native plant resources and local production, discussed further in the text. The plate represents a human community of consumers, and the squares represent their food resources. The area of each square represents its actual size and its relative contribution to the food supply of the consumers. A small square stands for a native plant that can supply food only when grown in its native range. The total area of squares is equal in all figures. **(A)** a community and its food demand; **(B)** a community reliant on four crops that each supply a quarter of the demand; **(C)** a community reliant on a high variety of plant resources, 25 times more diverse than community B; **(D)** a community fed by four plant resources, one of them is in proximity, the other three distant, demanding long chain of transports. Black arrows denote transport or food miles; **(E)** a community relying on one short-chain food resource and many small resources with long supply chains; **(F)** a mixture of one big resource in the vicinity of the community, two big and remote sources, and some small resources, most of them remote and two are local; **(G)** a community relying on a variety of locally grown plants.

Once a plant is identified as a novel food with good potential, its agricultural commercialization can be developed through two distinct strategies: one is establishing cultivated crops and the other is developing solutions for the efficient, cost-effective and ecologically sustainable gathering of native foods. Developing novel cultivated crops requires vast investments of time, knowledge, cash and patience for the long trial-and-error learning process that is required, which is why new crops are rare. Leaving the crop in its native habitat is a good solution, as illustrated globally with many plants. Coffee and cocoa—and to some extent tea, rice, coconut palm, avocado, date palm and pineapple—are examples of plants that are cultivated locally in their natural habitats and consumed globally. These systems challenge the concept of native plant use locally (see Figure 2): Is the global commercialization of a native cocoa plantation considered local food? Is it good for the local environment? We posit these extreme cases of native plant production, harvesting, transport, and consumption do not fit our thesis that promoting local food is neglected or necessarily beneficial. A related issue is use of native plants to improve existing conventional crops through back-to-nature crop breeding (Palmgren et al., 2015). This aspect is extensively studied and is not the focus of this review. Finally, natural systems are hard to mimic, and many species are impossible to domesticate. Yet, commercial use of wild plants can be economically plausible. Contemporary food gathering has great potential to expand the use of local plants, in concert with properly managing natural ecosystems, their resources and services, and improving plant gathering techniques at commercial scales.

## The benefits and assets of local food production and developing new crops from native plants

In the developing world, 10–15% of one billion hectares are farmed using traditional methods. Approximately 475 million people cultivate food in smallholder farms (FAO, 2016). Local production of food can reduce the carbon footprint of agriculture by lowering costs of production, shortening food miles, boosting local economies, and providing foods that are fresher and more nutritious for customers. The discipline of economic sociology links local food production to an increased sense of self-reliance or embeddedness of provisioning services, resulting in tighter social connectivity among individuals within communities and the landscapes they inhabit (Hinrichs, 2000). Indeed, in many industrial countries, the last decade saw proliferation of short-distance cooperative distribution and delivery programs such as community gardening and urban farming, farmers' markets, and various forms of community-supported agriculture including vegetable box delivery. These trends set the stage for native plants to develop into new biological resources that promote food diversity and crop resilience and enhance ecosystem services. The following is a more detailed list of the assets of local food production and utilization of native plants for food.

Advantages of practicing local food production:

1. Greater proximity of food production and consumption can lead to less waste and lower inputs of energy for transport, storage and preservatives, as well as support the recycling of plant nutrients, water and other inputs on site (for a review of the many inefficiencies in agriculture see Alexander et al., 2017). Local food supply helps to reduce food miles thus reducing carbon emissions (Cowell and Parkinson, 2003; Winter, 2003). For example, Coley et al. (2009) suggested that a round-trip distance of less than 6.7 km by each customer to purchase vegetables has a lower total carbon footprint than a system of regional storage and transport of the same food directly to the customer.
2. Locally grown crops supply fresher and potentially healthier food through reduced use of preservatives and reduced loss of nutritional value. Fresh food in short-chain production systems is less likely to be heavily processed. Processed food can negatively affect health by altering food preferences and appetitive states (Provenza et al., 2015).
3. Domestic production implies self-reliance with less imports (Little and Horowitz, 1987), which can promote societal sovereignty that may become essential if the availability and cost of fossil fuels make long-distance transport prohibitive. In addition, local production and delivery promote small-scale entrepreneurship, cultural diversity, sense of community, cultural and physiological relationships between people and seasonal availability of different foods.
4. At the ecosystem scale, the use of local plants can decrease the risk of exotic plant invasions that can adversely affect biodiversity (Cardinale et al., 2012). Compared with large-scale monoculture agriculture, local food production can reduce the spread of disease and the effects of invasive species. Transport infrastructure has an enormous impact on ecosystem fragmentation: the smaller the production-consumption circles, the smaller the impacts of fragmentation (Gehring and Swihart, 2003).

Advantages of using native plants and developing new crops:

1. Promoting genetic diversity. People have selected for growth over phytochemical richness in domesticated crops over the past 10,000 years (Provenza et al., 2015). In the process, domestication created a bottleneck of genetic diversity, as numerous genes were out selected (Vigouroux et al., 2005). Limited diversity of crops increases risks of disease and reduces potential for climate change adaptation. Native populations serve as a genetic bank that can enrich genetic diversity and phytochemical richness of crops, which in turn promotes resistance to adverse environmental conditions (Palmgren et al., 2015). The opportunity to develop and manage a greater array of native plants is critical to enhancing genetic diversity with potential for agricultural use.
2. Recruiting new local foods and crops is a way to diversify commercial uses, dietary options, and income for the local communities that rely on agriculture. Notably, the adaptation of local communities to climate changes will be critical for food security and poverty reduction (FAO, 2016).
3. Native plants are adapted to their homeland environment and thus better able to survive and produce high yields of phytochemically rich foods with fewer inputs including water, fertilization, and pest and disease control (Provenza et al., 2015).
4. Native plants are likely to mitigate soil erosion and conserve plant-microbe-soil interactions. Bacteria, fungi, endophytes and rhizobia in the rhizosphere are essential for health of plants and animals (Hawkes et al., 2007; Balestrini et al., 2015). These findings, which suggest we have underestimated the role of belowground interactions of plants with other organisms historically (Shelef et al., 2013), offer great potential to improve plant performance and crop yields (Drinkwater and Snapp, 2007). Mutualistic associations take time to arise. Therefore, an optimal holobiome—sum total of all genomes in a living system—will be easier to maintain in the plant-rhizosphere-soil continuum developed in the location of origin than in a mixture of soil, plant and other inputs derived from different and distant locations not locally adapted. Plant diversity can also be maintained in the context of a shared holobiome, representing not only the genetic variety of the individual plant genomes but also the metagenome including associated fauna, such as the microorganisms in the rhizosphere and the phyllosphere, which contribute to efficient plant growth under evolving environmental conditions (Pérez-Jaramillo et al., 2016). Agricultural management based upon a metagenomics perspective can help to protect against emerging plant diseases and pests, and can potentially reduce the use of hazardous pesticides. In addition, decomposition processes are likely to occur faster and more efficiently with the home field advantage of native soil, plants, and herbivores (Ayres et al., 2009).
5. Incorporating native food plants as temporal and spatial intercrops for land management can help to maintain soil quality and prevent soil degradation. The no-tillage strategy depends on the availability of appropriate plants, often the local plants found in the field. Intercropping also helps to maintain soil quality and enhance nitrogen uptake (Eaglesham et al., 1981), repel herbivores and other enemies (Tonhasca and Byrne, 1994), reduce weeds (Liebman and Dyck, 1993) and offer a higher net income to farmers (Yildirim and Guvenc, 2005). Local plants as intercrops have two prominent advantages—local adaptation is likely to occur with little external inputs of water or fertilizers and the hazard of invasive species is avoided by using noninvasive species.
6. By augmenting local food production with native plants, people can enhance the diversity and resilience of existing crops, using genetic diversity of native progenitors or crop-recent relatives that preserve desired traits. In tomato, for example, wild species outperformed the elite varieties for total yield and soluble solids (15%), and fruit color and sugars (40%), as compared to the normal improvement of 1% achieved annually through traditional breeding (Bernacchi et al., 1998). Similar potential exists for the wild type gene banks of the main crops (Tanksley and McCouch, 1997). Cox et al. (2006) discuss the benefits of breeding and domesticating perennial crops, including enhanced diversity of perennial plants in native terrestrial biomes as opposed to monocultures of annual crops. They also emphasize that today no perennial crops produce adequate grain yields, though the perennial crops that have been developed tend to store more carbon and require less resources. Science can expedite processes that a few millennia ago took centuries to develop, including improving food quality and resilience, and breeding perennial crops has been initiated in wheat, sorghum, sunflower and wheatgrass (Cox et al., 2006). Diamond (2002) stresses that knowledge regarding the control of bitterness and astringency will allow selection for fruits that were not edible before, for example acorns.
7. Local agriculture and native plants can help reduce human conflicts, diminish exploitation of labor forces in developing countries and enhance fair trade. An interesting example is the cassava market. The starchy roots of cassava (*Manihot esculenta)*, native to Brazil, were expanded to a global production of nearly 270 m tones a year by 2014 (FAO). This drought-tolerant crop is popular in small stakeholder farms in rural areas of Latin America, Asia and Africa, (Henry and Gottret, 1996). It is a unique example of a native Brazilian plant that is successfully cultivated and globally distributed, yet used primarily for self-production in short-chain markets. On the other hand, quinoa illustrates the problems that can occur when a local species is sold on international markets. Jacobsen (2011) argued that increased demand for quinoa put too much stress on the environment in Bolivia, leading to diminished biodiversity and land health. Quinoa illustrates the complexity of defining “local food” in a global economy. This crop is grown in its natural homeland, due to biological constraints, similar to many other crops including coffee, tea, cocoa, spices and herbs. Once commercialized and distributed throughout international markets, the impact on the local farmers can be uplifting or devastating. Nevertheless, we argue that with fair trade awareness and market incentives the use of native plants can expand and diversify agricultural resources.
8. Native species can reduce negative impacts of introduced species. Invasive species often spread and damage the environment, threatening biodiversity, agriculture, and human health (Schmitz and Simberloff, 1997). Insect outbreaks transform ecosystems (Foucaud et al., 2010); mammalian population outbreaks damage ecosystems and risk human safety (Côté et al., 2004); and weeds adversely impact rangelands across the U.S. and worldwide at an alarming rate (DiTomaso, 2000; Duncan et al., 2004). Recently, the EU Council adopted regulations on preventing and managing invasive species (PE-CONS 70/14, 13266/14 ADD 1), suggesting that of 12,000 alien species in Europe, as many as 10–15% spread and cause damage, estimated at 12 billion Euro each year. Clearly, encouraging the production and use of local species could help to alleviate these issues.
9. Using native species can positively influence human health. The so-called Western diet has changed key nutritional characteristics of human diets worldwide, especially with the introduction of processed foods. In addition, the food industry has selected for fruits and vegetables of low palatability by favoring varieties that are less phytochemically rich than their wild ancestors (Robinson, 2013; Reeve et al., 2016). Agricultural practices further diminish phytochemical richness by increasing resource availability through fertigation with off-farm sources of nitrogen, phosphorus, and potassium. Primary and secondary compounds increase when plants are mildly stressed due to less availability of nutrients and water, but decrease when agricultural practices emphasize productivity and growth (Bryant et al., 1983; Coley et al., 1985). Expanding and diversifying use of native plants, in combination with cultural practices for preparing those foods, would add health-promoting phytochemicals to diets and nullify the apparent economic costs of such practices (Provenza et al., 2015). The use of native plants, some of which have been used by humans for centuries, will result in vegetable foods that are highly nutritious, palatable and easily digested.

In summary, significant advantages accrue to using local plants to supplement food production, and through the phytochemical richness they possess, enhance human health (Provenza et al., 2015). In addition to enhancing diet diversity for people, enhanced use of local plants will diversify agricultural entrepreneurship and preserve genetic diversity so as to enhance crop endurance during stressful environmental conditions. Local species can reduce input investment and environmental conflicts. Even if local species are not economically relevant globally, maintaining a diversity of plants from different geographic regions is important locally. Diverse plant communities have myriad adaptations to environmental stressors, developed over thousands of years in response to adverse environmental conditions. Seed-bank collections can provide a genetic resource to grow plants in various environmental conditions in different geographic areas under changing climates (Dempewolf et al., 2014). Domestication of plants, one of the most influential processes in human history, resulted in vast socioeconomic improvements and human development. According to Harris and Hillman (1989), the main trends were increasing sedentism (settlement size and duration), population density, and social complexity from ranking to state formation. Domestication of new crops has nearly stopped, supplanted by plant varietal breeding (and genetic modification) of already domesticated species. This practice creates a genetic bottleneck. For example, the rich reservoir of wild tomato species has narrowed to a few genetically poor cultivated varieties of tomatoes (Bai and Lindhout, 2007). Miller and Tanksley (1990) estimated that less than 5% of wild tomatoes' genetic diversity is contained in the genomes of modern cultivars. The current presumption in research and practice is that agro-variability could be remunerated by introgression of adaptive traits from wild species to existing crops (Zamir, 2001) by researchers seeking to improve crop resistance to abiotic stress (Flowers, 2004; Tester and Bacic, 2005), disease (Johnson and Jellis, 2013), and herbivory (Chaudhary, 2013). With growing initiatives to improve agriculture through science and technology, expanding use of native plants as novel crops is calling for more attention. To do so, we must first learn the challenges of developing new crops. If the benefits of using local species outcompete the use of global crops, why are they not used more frequently? Here we present some of the main reasons.

## Obstacles to domesticating local plant species and commercializing their products

Despite the advantages, recruitment of new crops from native plants is extremely challenging. Several obstacles explain why relying on native plants to supplement our diets remains to be developed for the future, and is not yet a common practice:

1. Intensive agriculture selects for cash crops at the expense of developing new crops with lower environmental impacts. Existing crops are ready to use, whereas developing new crops is demanding and risky. Existing companies, families, machinery, roads and customers are all part of a well-known infrastructure for food production. Neither producers nor consumers are interested in leaving the familiar system to risk investing in new crops. Evolving from the familiar into the unfamiliar typically comes about only when people are under great duress (Massy, 2017).
2. Consumer acceptance of novel food is hard to predict. An interesting example is the acceptance of juice made of Açaí palm (*Euterpe oleracea*). The plant, native to Brazil and Trinidad, has a growing market as a healthy tropical juice commercially distributed in Europe and the USA. Sabbe et al. (2009) showed that consumer acceptance and purchase intention of the fruit juice was affected by interactions among many variables including socio-demographic characteristics, health-orientation, perception of health claim, and of course, to a large extent, taste experience. A rich body of literature is related to causes and consequences of “food neophobia,” the fear of eating unfamiliar foods (see for example Dovey et al., 2008).
3. Domestication depends on financial investment and has high risk. This implies that modern domestication can flourish only with the strong support of policy makers and people with strong financial interests.
4. Regulatory barriers exist for developing new crops. New foods require the approval of government agencies. Proving that a new food is safe for all consumers is not an easy task. Only a handful of countries (e.g., Australia, Britain, USA, and France) possess the technical and procedural abilities to assess the risks of eating new foods. Most governments rely on protocols and lists of edible species produced in those countries. If the new food is not on those lists, regulators are unlikely to prioritize investments in the risky process of developing new crops, resulting in missed opportunities for the entrepreneurial development of new crops derived from native plant species.
5. In some countries, the use of local species may give rise to intellectual property concerns (Ahmed and Johnson, 2000), as indigenous communities may claim local plants and cultivation and gathering procedures as their sole property.
6. Exploiting indigenous peoples' rights (Lee, 2013) may hinder domestication efforts. Indigenous communities tend to protect their resources, which can cause conflicts when other people want to share their experience. Cultivating food that was formerly collected in the wild may require careful analysis of the effects of the new practice on rural farmers and harvesters (Stewart and Cole, 2005). The surging economy generated by the Açaí palm, for example, has negatively impacted local communities in the Eastern Amazon estuary. The intensification of Açaí forestry impacted land tenure systems, transportation systems, and social inequalities among the local Caboblu producers due to the growing demand from international urban centers (Brondízio et al., 2002).
7. Risk of overexploitation. Souther and McGraw (2014) predicted that climate warming (1°C, next 70 years) and harvest will result in high risk of extinction of American ginseng (*Panax quinquefolius* L.). Similarly, local species are used in oil palm agriculture, but 60% of the oil palm plantation land use is at the expense of natural forests, threatening their unique biodiversity and many ecological services (Koh and Wilcove, 2008). Thus, the use of local species must involve a thorough study of the effects on ecosystems including species biology, carrying capacity and interactions with other species. Cultivating an over-harvested plant can provide strong conservation benefits while still providing food and income to indigenous populations, a strategy preferred by Tekinşen and Güner (2010), who study tubers of native Turkish orchids. The tubers of at least 30 species and 10 genera of the *Orchidaceae* family are traditionally collected to produce a local delicate hot drink known as “*Salep*,” as well as, among other products, a savory stabilizer of ice cream. This high-quality local plant product has been traded in the Mediterranean region for centuries. Nevertheless, producing 1 kg of *Salep* requires thousands of dried tubers and irresponsible plant poaching exposed the orchid population to the risk of extinction—an estimated annual damage to 120 million wild *Salep* plants (Kreutz, 2002).
8. Biological barriers to domestication. Only a handful of plants have been successfully domesticated in the last centuries. They include strawberries, blueberries, macadamia, and pecan nuts, which all had negligible economical value as compared to ancient domesticated plants. An interesting example is the enormous effort invested attempting to domesticate truffles. The desert truffle *Terfezia boundieri* is associated with the host plant *Helianthemum sessiliflorum* (Turgeman et al., 2011). For decades, local Bedouin people have eaten the truffle, which has great potential as a gourmet food, highly valuable nutritionally and commercially (Kagan-Zur et al., 2013). Truffles could be a novel crop with low inputs (Kagan-Zur, 2001). Nevertheless, the complex symbiosis of this mycorrhizal system (Zaretsky et al., 2006) has not proved easy to domesticate and commercialize, despite several decades of research. The same is true with huckleberries (Barney, 2003). Another example, among many others, is the desert plant *Erodium crassifolium*, an edible tuber plant used traditionally by indigenous peoples (Batanouny, 2001), which was never commercialized despite the fact it could potentially serve as an energy source (carbohydrates) and a low input crop.
9. In addition to plant biology, some agro-technical issues must be addressed, even when a plant is successfully transferred from its native habitat to an agricultural field. The quality and quantities of plant products are affected by seasonality, climate, temperature, soil, nutrients and water supply. For example, secondary metabolites of plants are often the target of cultivation, as in the case of spices, tinctures and drinks. However, the production of secondary metabolites can be significantly altered when nutrient and water supply is insufficient (Gershenzon, 1984), or with seasonal changes (Grulova et al., 2015). Hence, finding the best conditions to develop a new cultivar demands ample amounts of trial and error, meaning vast investment of time, labor and resources. Commonly, harvesting fruits and other plant parts from naturally occurring stands and trees is more practical than cultivation and domestication (Barney, 2003). However, some masting species like acorns are subject to long reproductive maturity and episodic fruit production.
10. The use of local varieties may result in the disappearance of cultivars that support regenerative agriculture. For example, Oriental Wheat Triticum turanicum Jakubz (Grausgruber et al., 2005) is praised as a highly nutritious pure ancient stand. Avoiding the use of this cultivar just because it has expanded far from its area of origin (Anatolia, according to Gökgöl, 1961) would have contradicted many other aspects of promoting regenerative agriculture.
11. Once established, a new crop could rapidly spread and would not be a local crop anymore. The direct consequence is that a successful new crop could inhabit new places and become a well-established exotic and potentially invasive harmful species. This can be avoided if plants are used in their native range. For certain crops such as coffee, rice, and certain tropical fruits, biological barriers dictate that crops are used only in their home ranges.

## Utilization of local plant species—the case of pine nut production in the western US

While export of agricultural products occurs globally, there are plenty of untapped local resources. For example, approximately 11 species of North American pinyon pine produce edible and highly nutritious nuts, with the most important being Colorado piñon (*Pinus edulis*), dominant throughout pinyon-juniper woodlands of the southwestern USA and Colorado Plateau, and singleleaf pinyon pine (*Pinus monophylla*), which is abundant throughout the Great Basin “cold desert” of Nevada and western Utah. Archeobotanical records have dated pine nut gathering in Utah to at least 7500 years before present (Rhode and Madsen, 1998). As climates warmed and some species moved north during the Holocene, the arrival of *P. monophylla* to the Great Basin approximately 6000 years ago provided a critical protein source that allowed people of the Middle Archaic period to extend their seasonal use patterns beyond the wetland habitats bordering pluvial lakes, into the surrounding uplands (Simms, 2008). Today, the same *Pinus* species cover large portions of western North America, estimated at approximately 56 million acres (Mitchell and Roberts, 1999), equivalent to 22.6 million hectares.

Although piñon pine nuts are more nutritious than many other tree nuts that are extensively cultivated in orchards—*P. edulis* is rich in oils and *P. monophylla* is rich in proteins and carbohydrates (Lanner, 1981)—pine nuts in the United States are harvested only locally and nut harvests are not commercially important. Yet large quantities of pine nuts are consumed each year in the United States, often serving as a key ingredient in pesto, salads and various Mediterranean dishes. Rich in unsaturated fatty acids, pine nuts are beneficial for controlling coronary heart disease through reduction of lipids in the circulatory system (Ryan et al., 2006). In a $100 million market over 80% of pine nuts consumed annually in the United States are imported mainly from eastern Asia (Russia and northeastern China; *Pinus koraiensis*) and Mediterranean Europe (*Pinus pinea*) (Sharashkin and Gold, 2004). As a result, massive collection of pine nuts in Russia and northeastern China continues to degrade the Korean pine broad-leaved forests (Ogureeva et al., 2012; Zhao et al., 2014), thousands of miles away from regions in North America and Europe where the nuts are consumed (Slaght, 2015).

Despite the advantages, developing a commercial, local pine nut industry in the western U.S. faces multiple challenges including:

1. Long generation time: reproductive maturity occurs at 25–50 years, with maximum seed production occurring at 75–100 years (Krugman and Jenkinson, 1974).
2. Episodic seed production: Good crop years of these masting species are highly variable in space and time, occurring every 4–7 years (Barger and Ffolliott, 1972). During drought periods, the frequency of good mast years can be reduced by as much as 40%, particularly when drought is associated with high late summer temperatures (Redmond et al., 2012).
3. Picking nuts is laborious work and access to nut-producing woodlands is often limited.
4. Potential competition with cultural users of pine nuts. Pine nut gathering remains important to native peoples in the region, and increased commercialization of the pine nut could come into conflict with such uses.
5. Potential ecological sustainability issues. Commercial pine nut harvesting could create competition for critical forage resources required by certain seed-caching wildlife species, including Pinyon Jay, Clark's Nutcracker, and several species of fossorial rodents (Vander Wall, 1997). Widespread seed harvesting could also negatively affect the regeneration potential of piñon pine populations, and hence resilience to episodic drought events that cause extensive overstory mortality (Redmond et al., in press).

Management of pinyon-juniper woodlands in the Western United States has not strongly considered the food value of pine nuts. In combination with recent drought events that have resulted in widespread tree mortality that threatens the long-term resilience of pinyon-juniper woodlands (Breshears et al., 2005; Redmond et al., in press), recent and planned management activities also threaten to reduce the availability of the pine nut resource. Pinyon-juniper woodlands are currently targeted for widespread tree removals across large areas of their distribution, particularly in the Great Basin. The objectives are to create forage for livestock and game mammals, to create or maintain habitat for sagebrush specialist species such as Greater Sage-Grouse, to provide woody fuels for bioenergy projects, to reduce fire risk, and to increase resilience to post-fire invasion of exotic annual grasses by fostering an understory of native perennial herbaceous species (Chambers et al., 2014). Ironically, extensive tree removal projects have occurred or are planned in many areas that were tree-dominated prior to Euro-American settlement, but were harvested in the late nineteenth Century to provide charcoal and woody fuels for mining-related activities (Young and Budy, 1979; Ko et al., 2011; Lanner and Frazier, 2011). Subsequent regrowth over the past 100–150 years is commonly viewed as an expansion of tree cover by human inhabitants of the region, whose generation time is much shorter than that of pinyon pines. In any case, many of the desired management objectives for fire risk reduction and conservation of understory plant species and the associated shrub-steppe habitats do not require complete woodland removal, and can be compatible with the goal of maintaining abundant pinyon pine seed production for wildlife and humans. Silvicultural methods, likely including uneven-aged management on favorable sites, can be further developed to promote drought-resilient, fire-resistant woodlands with a significant proportion of seed-producing trees (Gottfried and Severson, 1993; Page, 2008). Cone production in *Pinus pinea* can be increased by judicious thinning (Moreno-Fernandez et al., 2013).

One requires only a small stretch of the imagination to envision people in the Western United States meeting their demand for pine nuts through purchase from local harvesters, or by harvesting the nuts themselves when cones ripen in the autumn. This would greatly reduce the carbon footprint associated with pine nut importation, and would require no water use or fertilizer application, as piñon pines occur naturally under the driest conditions and in relatively nutrient-poor soils. Increased consumption of locally harvested pine nuts might also have the desirable effect of reducing the incidence of “pine nut syndrome” or “pine mouth”. This condition is characterized by an annoying metallic taste that can linger in the mouth for multiple days, and that has been associated with consumption of *Pinus armandii*, an inedible pine species whose nuts are occasionally found mixed within pine nut batches that have been imported from Asia (Mikkelsen et al., 2014).

Despite all the good reasons, economic and environmental, to promote a local agriculture of pine nuts, we are still far from seeing considerable change from importing these nuts to developing local production. In a world motivated by short-term economic incentives, with nearly unlimited transportability of foods across the globe, most foods people eat are not produced locally. If costs for transport increase, due to rising costs of fossil fuels, that will drastically change the value of local food production and consumption.

## Future prospects of local food production

A recent call to rethink the research and development of food production urges us to nourish humanity more efficiently and improve the food disparity of a world in which 795 million people are undernourished and 2 billion adults are overweight or obese (Haddad et al., 2016). Haddad et al. (2016) discuss ten global research goals, two of which are closely related to our discussion. The first implies understanding the role of food-chain length. Ultimately, that would lead to an optimal mix of short-chain systems where high-quality food is produced and consumed nearby and long-chain systems where large quantities of food travel great distances (see Figures 2F,G). Second, they argue that to improve global food production we must analyze business incentives, mainly for private farmers, retailers and food processors. To help kick-start these activities, we contend that governments should offer more incentives for shorter food-chains by finding solutions to enhance diversity of uses of native plants. Awareness of consumers and farmers for the benefits of commercializing native species will play an important role. The local food movement, urban farming, production and consumption of pesticide-free healthy, nutritious, savory and sustainable food have attracted a great deal of attention in the last decade.

We refer here to agriculture as a more complex system than traditional cultivated crops. Agriculture has a strong impact on the environment: soil and water quality and quantity, deforestation, habitats and biodiversity, intensive farming, economic and social conditions in rural communities (Massy, 2017). The consequences can include the loss of biodiversity, accelerated land degradation, high fertilizer inputs, water contamination and the spread of pesticides hazardous to human health. Regenerative agriculture has arisen as a reaction to the negative effects of agriculture including impacts on land and resource management, humans and ecosystem interfaces. Agricultural practices can move from external-input farming to low-input practices (e.g., water, nutrients, pest control, land, energy) without significantly reducing production (Pittelkow et al., 2015). One of the greatest challenges for agriculture is to reduce the distances between crop production and food consumption. In some cases, this challenge can be met by using local species.

Recruiting native plants to develop cultivation of novel crops has great potential to establish new markets. This potential is countered by great challenges and enormous financial demands—lack of knowledge concerning unfamiliar species, the need for hybridization and agro technical improvements, sometimes with slow growing plants, and the risks associated with exchanging existing crops for uncertain income opportunities in an already conservative market. Some plant species are completely incompatible with any sort of domestication, or their cultivation requires an enormous investment of research, time and money. That is the case for slow growing species (e.g., many trees), plants with specific and narrow niche breadth (e.g., orchid tubers), and food sources that require complex biological interactions that are hard to mimic (i.e., edible mycorrhiza). Nevertheless, the success of some plants that are now harvested for commercial use (e.g., truffles, pine nuts, berries, spices, and herbs) demonstrate that modern food gathering is feasible. Food gathering may be improved in various ways, although many of them are not commonly practiced and deserve more attention. The first step is developing tools to find biological resources that are not used today, by expanding the strategy of ethnobotany, with its pros and cons. People also must continue to evolve ways to better manage naturally occurring plantations, a process that is site-specific. The last step is improving technological solutions for gathering, picking and processing wild fruits and other plant organs. Commercial gathering and developing new crops may balance each other, as the risk of overexploitation may be offset by mitigation of undesired plant invasions and overuse of agricultural inputs.

Local does not necessarily mean native, and using non-native foods grown, harvested, stored and delivered near the place of their consumption is advantageous. Native plants can complement these efforts. Native plants require lower inputs of water, nutrients, pest control and energy. Nevertheless, the long road to greater use of native species and local food production has many obstacles to overcome. Biological barriers to domestication are a challenge. In addition, global markets make it difficult to establish new crops. Other barriers include lack of financial incentives and investments, regulations, and agro-technical boundaries. Moreover, a successful new crop is likely to spread rapidly across the globe, losing its local value. Despite these challenges, the advantages of using native plants for food production are many. They include enabling diverse agriculture entrepreneurship, preserving interspecies crop and genetic diversity to enhance crop endurance in adverse environmental conditions, reducing inputs, reducing conflicts over indigenous land management, reducing environmental conflicts, and intercropping to improve land management.

## Conclusion

To date, most research and practical efforts have been devoted to improving existing crops, rather than recruiting new, local species. We conclude that native food production should receive more attention in research and application to initiate and empower regenerative agriculture. Moving from monocultures to more diverse local crops, and domestication of new species, can conserve biological resources, and help to foster more sustainable agroecosystems. However, the use of native plants in local food production has not yet attained a high level of awareness. To reach an optimal balance between short- and long-chains of food production, shorter chains should be supported more vigorously and the evaluation of this balance should consider a more thorough-life-cycle analysis of food production (Edwards-Jones et al., 2008). A pivotal strategy to support more local sources of food production is to allocate more resources for improving harvesting of local plants.

## Author contributions

All authors have made a substantial, direct and intellectual contribution to the work, and approved it for publication. OS initiated the work, PW elaborated on the case study of pine nuts, FP was a pivoting writer and improved articulation.

### Conflict of interest statement

The authors declare that the research was conducted in the absence of any commercial or financial relationships that could be construed as a potential conflict of interest.
